# Analyzing anatomy over three dimensions unpacks the differences in mesophyll diffusive area between sun and shade *Vitis vinifera* leaves

**DOI:** 10.1093/aobpla/plad001

**Published:** 2023-01-25

**Authors:** Guillaume Théroux-Rancourt, José Carlos Herrera, Klara Voggeneder, Federica De Berardinis, Natascha Luijken, Laura Nocker, Tadeja Savi, Susanne Scheffknecht, Moritz Schneck, Danny Tholen

**Affiliations:** University of Natural Resources and Life Sciences, Vienna, Department of Integrative Biology and Biodiversity Research, Institute of Botany, 1180 Vienna, Austria; University of Natural Resources and Life Sciences, Vienna, Department of Crop Sciences, Institute of Viticulture and Pomology, 3430 Tulln an der Donau, Austria; University of Natural Resources and Life Sciences, Vienna, Department of Integrative Biology and Biodiversity Research, Institute of Botany, 1180 Vienna, Austria; University of Natural Resources and Life Sciences, Vienna, Department of Crop Sciences, Institute of Viticulture and Pomology, 3430 Tulln an der Donau, Austria; University of Natural Resources and Life Sciences, Vienna, Department of Integrative Biology and Biodiversity Research, Institute of Botany, 1180 Vienna, Austria; University of Natural Resources and Life Sciences, Vienna, Department of Crop Sciences, Institute of Viticulture and Pomology, 3430 Tulln an der Donau, Austria; University of Natural Resources and Life Sciences, Vienna, Department of Integrative Biology and Biodiversity Research, Institute of Botany, 1180 Vienna, Austria; University of Natural Resources and Life Sciences, Vienna, Department of Integrative Biology and Biodiversity Research, Institute of Botany, 1180 Vienna, Austria; University of Natural Resources and Life Sciences, Vienna, Department of Integrative Biology and Biodiversity Research, Institute of Botany, 1180 Vienna, Austria; University of Natural Resources and Life Sciences, Vienna, Department of Integrative Biology and Biodiversity Research, Institute of Botany, 1180 Vienna, Austria

**Keywords:** Grapevine, intercellular airspace, leaf anatomy, microCT, photosynthesis

## Abstract

Leaves grown at different light intensities exhibit considerable differences in physiology, morphology and anatomy. Because plant leaves develop over three dimensions, analyses of the leaf structure should account for differences in lengths, surfaces, as well as volumes. In this manuscript, we set out to disentangle the mesophyll surface area available for diffusion per leaf area (*S*_m,LA_) into underlying one-, two- and three-dimensional components. This allowed us to estimate the contribution of each component to *S*_m,LA_, a whole-leaf trait known to link structure and function. We introduce the novel concept of a ‘stomatal vaporshed,’ i.e. the intercellular airspace unit most closely connected to a single stoma, and use it to describe the stomata-to-diffusive-surface pathway. To illustrate our new theoretical framework, we grew two cultivars of *Vitis vinifera* L. under high and low light, imaged 3D leaf anatomy using microcomputed tomography (microCT) and measured leaf gas exchange. Leaves grown under high light were less porous and thicker. Our analysis showed that these two traits and the lower *S*_m_ per mesophyll cell volume (*S*_m,Vcl_) in sun leaves could almost completely explain the difference in *S*_m,LA_. Further, the studied cultivars exhibited different responses in carbon assimilation per photosynthesizing cell volume (*A*_Vcl_). While Cabernet Sauvignon maintained *A*_Vcl_ constant between sun and shade leaves, it was lower in Blaufränkisch sun leaves. This difference may be related to genotype-specific strategies in building the stomata-to-diffusive-surface pathway.

## Teaser

Leaves developing in bright sunlight are markedly different from those developing in shade. Using 3D imaging techniques and a new approach for anatomical analysis, we studied sun and shade leaves of grapevine cultivars in detail. We found that cell shape, size and density work in tandem to increase the internal surface area of sun leaves relative to that of shade leaves, allowing for more efficient CO_2_diffusion inside the leaves and higher photosynthetic rates under high light. We also describe modular structures that we name ‘vaporsheds’, connecting the pores on the leaf surface (stomata) to the nearest photosynthesizing cells.

## Introduction

Light availability is generally heterogeneous within a plant canopy, with some leaves being exposed to direct sunlight and others being in various levels of shade. Light, absorbed by leaves, provides the energy for the assimilation of carbon dioxide (CO_2_) in the process of photosynthesis. The amount of carbon gained by photosynthesis per unit invested resources (i.e. photosynthetic efficiency) is affected by the morphology and anatomy of the leaves. Moreover, leaves modulate their structure based on the prevailing light intensities, resulting in higher photosynthetic efficiency (Givnish, 1988; Roderick *et al.*, 1999; Evans and Poorter, 2001). For instance, leaves grown at high light intensities (sun leaves) are typically thicker, more dense, and have a larger mesophyll volume fraction and cell surface area than leaves grown at lower light intensities (shade leaves, Poorter *et al.*, 2019). The higher density of sun leaves may be related to more vascular tissue, non-structural carbohydrates or thicker cell walls, but can also be related to the lower airspace fraction that has been reported for sun leaves (Hanba *et al.*, 2002; Tosens *et al.*, 2012; Fini *et al.*, 2016; Poorter *et al.*, 2022). Additionally, the shape of the cells in the mesophyll may differ, with the volume fraction of palisade tissue being higher in sun leaves, brought about by more palisade cell layers or by longer palisade tissue cells (Poorter *et al.,* 2019).

Despite the large differences in leaf thickness and density, light absorptance is typically similar between sun and shade leaves, as it is mainly dependent on chlorophyll content per unit leaf area, which is not much affected by the light environment (Evans and Poorter, 2001; Poorter *et al.*, 2019). However, photosynthesis of a shade leaf quickly becomes light saturated at higher light intensities, whereas a sun leaf is able to use more of the absorbed energy by distributing it over a larger number of chloroplasts within the leaf (Evans, 1999). Leaf anatomy plays an important role in this distribution of light intensity within the leaf, as shown by the low photosynthetic rates observed for sun leaves that were illuminated from the lower (spongy mesophyll) side (Evans and Vogelmann, 2003; Théroux-Rancourt and Gilbert, 2017). A large number of chloroplasts allows for an increased number of photosynthetic enzymes per unit leaf area, which can explain the higher rates of photosynthesis observed in thick sun leaves. But without increasing the surface area of the mesophyll cells, such an increase in biochemical capacity would make photosynthesis more limited by the slow CO_2_ diffusion across the cell walls (i.e. the liquid phase mesophyll conductance, Evans, 1999; Terashima *et al.*, 2001, 2011). Thus, the increased thickness of sun leaves can be seen as a feature that allows for a larger mesophyll surface area per unit leaf area, and thus higher photosynthetic capacity under high light (Nobel, 2009; Terashima *et al.*, 2011).

Although leaf traits can affect the absorption and penetration of light, differences between sun and shade leaves may not all be directly related to photosynthesis (e.g. high porosity and mechanical stability, Onoda *et al*. 2008). A further complication with analyzing the causes underlying differences in leaf traits is that they typically correlate with each other (Wright *et al.*, 2004; Shipley *et al.*, 2006). If such correlations are the result of architectural constraints, this can lead to incorrect attribution of functionality to features that arise out of structural requirements (Gould and Lewontin, 1979). Explicitly accounting for interactions between traits would provide a more integrated view and insight into how different characteristics of the anatomy work in tandem to bring about a functional change. For example, net photosynthesis and the conductance to CO_2_ have been shown to scale with the mesophyll surface area exposed to the intercellular airspace per leaf area, *S*_m,LA_, commonly abbreviated as *S*_m_ (Evans, 1999; Hanba *et al.*, 1999; Peguero-Pina *et al.*, 2017). This trait may be under selective pressure (Chatelet *et al.*, 2013) and it is well known that thicker leaves allow for a larger *S*_m,LA_ (Terashima *et al.*, 2001). However, larger *S*_m,LA_ can also result from other changes in the leaf anatomy, such as a reduced cell size or changes in cell shape (Terashima *et al.*, 2001; Chatelet *et al.*, 2013; Lundgren and Fleming, 2020; Théroux-Rancourt *et al.*, 2021).

*S*_m,LA_ can be understood as the product of several other phenotypic leaf traits that have been commonly analyzed in studies comparing sun and shade leaves **[see ****Supporting Information—Fig. S6** for more details**]**:


$$\begin{array}{l} S_{m,LA}=S_{m,Vcl}\left(1-\theta_{ias}\right)f_{mes}L_{leaf} \end{array}$$ (1)

where *S*_m,Vcl_ represents the air exposed surface to volume ratio of the mesophyll cells, θ_ ias_ is the airspace fraction within the mesophyll, *f*_mes_ is the volume fraction of the mesophyll in the whole leaf (i.e. mesophyll cells and the intercellular airspace) and *L*_leaf_ is the leaf thickness (see Table 1 for a list of abbreviations used). Equation (1) shows that the surface area available for diffusion can be enlarged by increasing the surface to volume ratio of the mesophyll cells (e.g. by decreasing cell size or modifying cell shape). *S*_m,LA_ can also be enlarged by reducing the airspace fraction in the mesophyll, increasing the amount of leaf volume allocated to the mesophyll or by increasing leaf thickness. Leaves may adjust any or all of these four traits to make more surface area available for diffusion. Since each of these traits can also be under selective pressure unrelated to CO_2_ diffusion, investigating the relative contribution of thickness, porosity, mesophyll fraction, and cell shape to differences in mesophyll surface area allows for a more fine-grained discussion of the acclimation of leaf anatomy to growth light conditions. Teasing apart the relative contribution of different anatomical characteristics to the area available for CO_2_ diffusion is a timely topic given the continued interest in improving photosynthesis through manipulation of mesophyll development (Terashima *et al.*, 2011; Tholen *et al.*, 2012; Lehmeier *et al.*, 2017; Ren *et al.*, 2019; Baillie and Fleming, 2020; Lundgren and Fleming, 2020).

**Table 1.** Table of abbreviations used in the main text.

Variables	Units	Definition
*A*_area_	µmol m^−2^ s^−1^	photosynthesis per leaf area
*A*_mass_	µmol g^−1^ s^−1^	photosynthesis per leaf dry mass
*A*_*V*_	mol m^−3^ s^−1^	photosynthesis per volume (see below)
*d*_pal_	µm	palisade cell diameter
*D*_pal_	mm^−2^	palisade cell packing density
*f*_mes_	µm^3^ µm^−3^	fraction of mesophyll volume within the whole leaf volume
*f*_pal_	µm µm^−1^	fraction of palisade thickness within the mesophyll thickness
*g*_ias_	mol m^−2^ s^−1^	intercellular airspace conductance
*g*_liq_	mol m^−2^ s^−1^	liquid phase conductance
*L*_leaf_, *L*_mes_	µm	leaf and mesophyll thickness
LA	cm^2^	leaf area
*S*	µm^2^	surface area
*S*_m_	µm^2^	surface area of the mesophyll cells exposed to the intercellular airspace
*S*_m,LA_	µm^2^ µm^−2^	*S*_m_ per leaf area
*S*_m,V_	µm^2^ µm^−3^	*S*_m_ per volume (see below)
*S*_m,vap_	µm^2^ µm^−3^	*S*_m_ per vaporshed
θ _ ias_	µm^3^ µm^−3^	mesophyll porosity
*V*_cl_	µm^3^	volume of the mesophyll cells
*V*_ias_	µm^3^	volume of the intercellular airspace
*V*_mes_	µm^3^	volume of the mesophyll (cells + intercellular airspace)
*V*_ias,vap_	µm^3^	volume of intercellulair airspace per vaporshed

**Figure 1.**
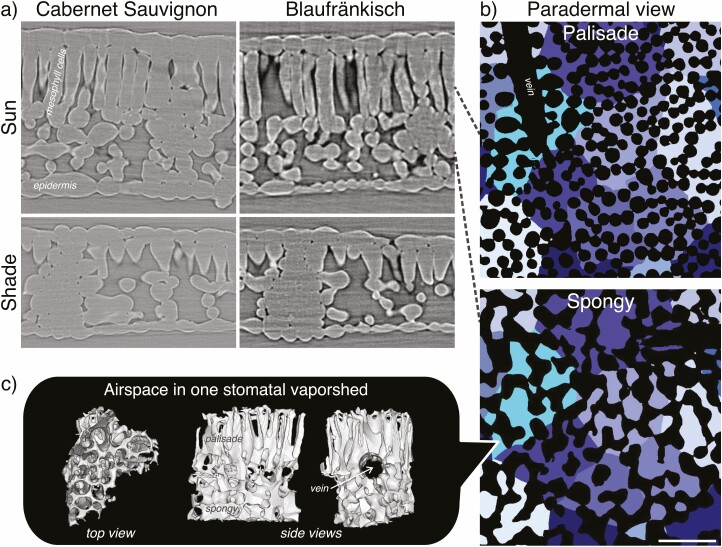
(a) Leaf microCT cross sections of Cabernet Sauvignon and Blaufränkisch plants grown under sun and shade conditions. (b) A paradermal section from a Blaufränkisch sun leaf through several stomatal vaporsheds. Each vaporshed has been labeled with a unique color (with mesophyll cells in black), and shows that vaporsheds slightly change shape from the palisade mesophyll (top right) to the spongy mesophyll (bottom right). (c) 3D representations of the airspace component of one stomatal vaporshed as seen from the top through the palisade mesophyll or from the side. Gray objects represent intercellular airspace pores. Airspace is absent from a cylindrical volume within the vaporshed where a vein was present (see also the paradermal view of the palisade in b). Cross sections and paradermal views are to scale (scale bar at the bottom right: 50 µm).

Since the leaf surface is not very permeable to water and CO_2_, the discrete nature of the stomatal pores produces lateral CO_2_ gradients across the leaf (Pickard, 1981; Parkhurst, 1994; Pieruschka *et al.*, 2006; Morison *et al.*, 2007). The spacing between the stomata is influenced by the growth light intensity (Muir, 2018) and contributes to the length of the diffusion path through the intercellular airspace, and thus also to the total CO_2_ diffusion resistance. As a result, structural traits affected by light intensity influence both the diffusion path length through airspace and the available surface area for the liquid phase conductance (*S*_m,LA_). To give appropriate weight to stomatal, airspace and liquid phase components the amount of surface area available for diffusion (and volume available for biochemistry) expressed per stoma becomes a necessary unit of comparison. We name this novel concept a ‘stomatal vaporshed,’ a 3D structure which, like a hydrological watershed, ‘drains’ vapor out of the leaf and acts as the smallest anatomical unit of the intercellular airspace (Fig. 1). For a typical vaporshed within a hypostomatous leaf, long branches of intercellular airspace with small diameter within the palisade connect to several larger volumes within the spongy mesophyll, pouring down into the substomatal cavity and finally out through the stomatal pore.

We set out to describe the relationship between 3D anatomy and the potential for carbon assimilation in sun and shade leaves of two grapevine (*Vitis vinifera* L.) cultivars. Light levels within dense grapevine canopies can drop below 1 % at some locations in the inner canopy (Smart, 1985), but even a moderate reduction (50–60 %) in the light availability results in marked differences in stomatal conductance, photosynthetic rates and leaf anatomy (Schultz *et al.*, 1996; Vanden Heuvel *et al.*, 2004), and has led to managing the access of leaves to light in vineyards (Smart *et al.*, 1982; Cartechini and Palliotti, 1995; Dokoozlian and Kliewer, 1995; Palliotti *et al.*, 2000; Chavarria *et al.*, 2012; González *et al.*, 2021). Instead of focusing on how individual traits, such as thickness or porosity, vary depending on the light environment, we analyzed their contribution to a functional trait: the surface area available for diffusion. We hypothesized that not only the leaf thickness, but also cell shape, leaf porosity, and investment in mesophyll tissue make a contribution to variations in the amount of diffusive surface per unit area or per stoma between sun and shade leaves. Leaf anatomy was imaged using microcomputed tomography (microCT) to capture all three dimensions non-invasively. We related anatomical measurements to leaf photosynthesis and discussed the implications of leaf construction strategies for carbon assimilation.

## Methods

### Plant material and growth conditions

The experiment was carried out over two consecutive years, in 2018 and 2019, at the facilities of BOKU UFT (Tulln, Austria). In the first year, rooted grafts of *Vitis vinifera* ‘Cabernet Sauvignon’ (clone 191E) on 101–14 rootstock (hereafter named CS) were acquired from a local nursery (*Reben Iby*, Neckenmarkt, Austria). In the second year, rooted grafts of *Vitis vinifera* ‘Blaufränkisch’ (clone 13-3 GM) on 5 BB rootstock (hereafter named BF) were acquired from the same nursery. Blaufränkisch is known to have originated from Lower Styria (present day Styria, Slovenia, Maul *et al.*, 2016) and to be genetically different from Cabernet Sauvignon (Magris *et al.*, 2021), which originated in the Bordeaux region in France.

The rooted grafts were planted in 7-L pots and allowed to grow in a glasshouse without any environmental control. For CS, nutrient-rich, sieved vineyard soil mixed with perlite (3:1 ratio) was used, while for BF pots were filled with commercial pot substrate containing slow release fertilizer (10 g pot^−1^, 15-5-20 NPK ‘Entec vino’). Pots were watered to pot capacity automatically every day. Clones were planted June 1 and April 1 in the first and second year, respectively. When all the plants had at least three mature leaves on one shoot, plants were pruned so that only one dominant shoot remained. To ensure that only leaves fully developed under different light conditions were used for further analyses, the last developing leaf below the tip was marked before moving half of the plants to the shaded environment (on June 29, 2018 for CS and on April 18, 2019 for BF). For the shade treatment, a tent of about 2 m (height) × 1.5 m (width) × 1.5 m (depth) was made from black polypropylene cloth (HaGa-Welt GmbH & Co. KG, Elze, Germany), resulting in a 60 % reduction in photosynthetic photon flux density (PPFD). A spectrometer (FLAME-S-VIS-ES, Ocean Optics Inc. Largo, USA) was used to confirm that under both light conditions, relative differences in the contribution of red, green blue and far-red light to the total PPFD were below 5 % (i.e. spectrally neutral shade).

During the week before synchrotron microCT scanning (last week of August in 2018 and first week of September in 2019), one mature leaf per plant was selected at least three leaves above the previously mentioned mark indicating the last developing leaf at the start of the shade treatment. The measured leaves were estimated to be about one month old, resulting in an average daily light integral (DLI) of 30 (CS sun), 12 (CS shade), 24 (BF sun), and 10 (BF shade) mol m^−2^ day^−1^. These estimates were computed using solar radiation measured at a weather station a few meters from the glasshouse, and using PPFD values measured inside the glasshouse. Average daily PPFD was below 700 µmol m^−2^ s^−1^ under full light, with maximum recorded values at leaf level of ~1200 µmol m^−2^ s^−1^ under full light and ~500 µmol m^−2^ s^−1^ under shade, i.e. ~60 % reduction.

### Gas exchange characterization

Selected mature leaves were characterized to assess their gas exchange properties using an LCPro-SD (ADC BioScientific Ltd., Hertfordshire, UK) gas analyzer equipped with a 6.25 cm^2^ leaf chamber. Leaves were light-acclimated in the gas-exchange chamber until stomatal conductance was above 60 mmol m^−2^ s^−1^ (but at least for 15 min) under ambient conditions (36 (SD: 2) °C and 55 (SD: 6) % RH). Net assimilation rates (*A*_area_) at a flow speed of 200 µmol s^−1^ and 1000 µmol m^−2^ s^−1^ PPFD was recorded. Light saturation for photosynthesis in field grown grapevines has been shown to occur at PPFD of 700–1000 µmol m^−2^ s^−1^ (Escalona *et al.*, 2000; Zufferey *et al.*, 2000; Cartechini and Palliotti, 2001).

### MicroCT scanning and image preparation

Leaf anatomy has typically been analyzed by light microscopy of thin transverse sections. Although it is relatively accurate to estimate traits such as lamina thickness and airspace fraction from a few sections, estimates of surface areas and volumes are prone to bias (Ivanova and P’yankov, 2002; Kubínová *et al.*, 2017; Théroux-Rancourt *et al.*, 2017; 2020b). In this study, we used microcomputed tomography to visualize a large amount of cells and airspaces in living leaves (Lehmeier *et al.*, 2017; Théroux-Rancourt *et al.*, 2017; Earles *et al.*, 2019). However, this method cannot image organelles, which prevents us from getting information on chloroplast position along the mesophyll cell surface for example (see Harwood *et al.*, 2021, for such a method).

Plants were brought to the TOMCAT tomographic beamline of the Swiss Light Source at the Paul Scherrer Institute (Villigen, Switzerland) in pots (CS) or as cut shoots (~1 m long) with the cut end placed in water (BF; cut 48 to 72 h before scanning). All of the plants for which photosynthesis had been measured were imaged, i.e. six leaves were scanned per cultivar and treatment, except for BF shade plants for which only five were imaged. Before scanning, a leaf was cut from the stem and a thin strip of ~1.5 mm width and 1.5 cm length was cut in between apparent higher order veins and immediately wrapped in polyimide tape as described in Théroux-Rancourt *et al.* (2017). Three (CS) or two (BF) strips were cut at different locations on the leaf surface to ensure within-leaf replications and to get better leaf-level averages, which are rarely available in microCT leaf anatomy data (Théroux-Rancourt *et al.*, 2021). Each strip was scanned within 15 min of being prepared by imaging 1801 projections of 100 ms under a beam energy of 21 keV and magnified using a 40× (CS) or 20× (BF) objective, yielding respective final voxel sizes of 0.1625 µm (field of view: ~416 × 416 × 312 µm) and 0.325 µm (field of view: ~832 × 832 × 624 µm). In the first year, a 40× objective was used, but later processing of the images showed no significant effects of downscaling the images by 50 %. We decided to scan using a 20× in the following year to reduce storage and computing costs. This magnification is higher than what is generally published, and using different magnifications has been shown to provide comparable trait values (Théroux-Rancourt *et al.*, 2021).

Using TOMCAT’s in-house reconstruction platform, scanned projections were reconstructed to cross sectional view using both absorption reconstruction (Marone and Stampanoni, 2012) and phase contrast enhancement (Paganin *et al.*, 2002), both of which are required for an accurate automatic segmentation of the images (Théroux-Rancourt *et al.*, 2020a). After aligning the stacks to be parallel to the image edges using ImageJ (Schneider *et al.*, 2012), at least nine slices were hand labelled using a graphics pen display tablet (Wacom Cintiq Pro 16) to precisely segment the background, the epidermis, and the vasculature. The mesophyll cells and the intercellular airspace were segmented by thresholding each absorption and phase contrast scan to maximize airspace volume (background) and taking care to avoid false segmentation within the cells (i.e. false segmentation of airspace). Any remaining false labelling of airspace or mesophyll cells was manually corrected. The hand labelled slices were then used to automatically segment the whole stack using a Python-based random-forest machine learning approach (Théroux-Rancourt *et al.*, 2020a).

### Leaf traits analysis of microCT images

**Figure 2.**
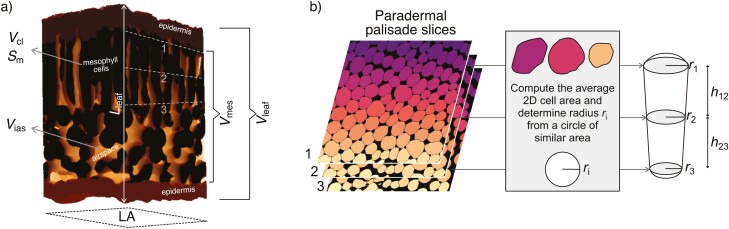
Visual summary of the methodology to extract anatomical data from microCT scans. (a) a 3D view of the leaf including mesophyll and epidermis. Symbols indicate leaf thickness (*L*_leaf_), leaf area (LA), mesophyll surface area exposed to intercellular spaces (*S*_m_), total leaf (*V*_leaf_), mesophyll (*V*_mes_), mesophyll cell (*V*_cl_) and airspace (orange, *V*_ias_) volume. After segmentation of such an image stack, volumes were determined by summing pixels in each slice and *S*_m_ was computed using a marching cube algorithm. (b) Palisade cell traits are computed by extracting three slices from the palisade tissue (1, 2 and 3) and applying a watershed algorithm to those slices to label individual cells. Analysis of the cross sectional area of these cells allowed for the computation of an average cell radius. The estimates for the cell radius (*r*_i_ at three different positions in the palisade tissue was used to compute an average palisade cell volume (*V*_pal_) and surface area (*S*_pal_), assuming the cells can be described using two juxtaposed truncated cones (right). Height *h* represents the length between the selected paradermal slices. Further details are presented in the Methods section.

Thickness (*L*) of the leaf, the mesophyll and each epidermis was computed as the median of all of the voxel columns along the leaf area within each scan (width × number of slices of the stack; *>*2 × 10^6^ values). Tissue volume (*V*) was extracted as the sum of voxels for each tissue: epidermis, vasculature (incl. bundle sheaths), mesophyll cells (*V*_cl_) and airspace (*V*_ias_). Mesophyll volume (*V*_mes_) was computed as the sum of *V*_cl_ and *V*_ias_. Surface area of the mesophyll cells exposed to intercellular airspace (*S*_m_) was computed through a marching cube algorithm (van der Walt *et al.*, 2014) building surface meshes around the airspace using a step size of two (i.e. over every second voxel), which provides a surface area estimate for geometrical objects closer to their mathematical surface area than when using a step size of one (Théroux-Rancourt *et al.*, 2020b). Mesophyll porosity (θ_ ias_) was computed as *V*_ias_/*V*_mes_. Stomata were only present on the abaxial side. Ratios of lengths, surfaces, and volumes were computed for each scan (e.g. the *S*_m_ ratios, θ_ ias_) separately, and an average was computed for each leaf using values from the two (BF) or three (CS) scans available. Thickness, surface and volume traits extracted from microCT scans are presented in Fig. 2a.

The methods previously described provide tissue-level data but do not inform us on cell-level properties. Getting a large amount of 3D single cell data is computationally challenging and prone to misclassification, hence previously published data were generally acquired using either limited 3D datasets (Théroux-Rancourt *et al.*, 2020b; Harwood *et al.*, 2021) or destructive methods (Ivanova and P’yankov, 2002). Using 2D slices of microCT scans benefits from the non-invasive nature of the imaging method, i.e. minimal disturbance of the leaf sample, while allowing for the acquisition of large amounts of 2D values (Théroux-Rancourt *et al.*, 2021; Borsuk *et al.*, 2022). To extract data on palisade cell properties, three 2D paradermal slices of a region of the scan containing only mesophyll cells were selected: one at the first position fully into the palisade below the adaxial epidermis, a second at the very bottom of the palisade before the spongy mesophyll, and a third one exactly in the middle between those two. The pipeline used to estimate 3D palisade traits from three 2D slices is visually presented in Fig. 2b. First, a watershed segmentation was done on each 2D slice to identify every individual cell present (*>*100) using the MorphoLibJ package of ImageJ (Legland *et al.*, 2016). Palisade cell density within the mesophyll tissue was computed as the number of cells per area of the image, excluding cells touching the left and bottom edges. Single cell projected area at each measured position along the palisade was then extracted using the Scikit-Image Python package (van der Walt *et al.*, 2014). The diameters (*d*) of the cells completely enclosed in the image (i.e. not touching the edges of the image) were then estimated from a disk of equivalent area (*A*), such that $d=2\times r=2\times \sqrt{A/\pi }$, where *r* is the radius. Single cell volume was then estimated from the volume of two truncated cones between the three measured slices:


$$\begin{array}{l} V_{pal}=\frac{\pi h_{ab}\left(r_{a}+r_{b}+r_{a}r_{b}\right)}{3} \end{array}$$ (2)

where *V*_pal_ is the volume of one truncated cone, *h*_ab_ is its height, i.e. the distance between the respective measured slices, and *r*_a_ and *r*_b_ are the radii of the top and bottom surfaces. The lateral surface area for individual palisade cells (*S*_pal_) was estimated from the lateral area of a truncated cone, such that:


$$\begin{array}{l} S_{pal}=\pi \left(r_{a}+r_{b}\right)\sqrt{h_{ab}^{2}+{\left(r_{a}+r_{b}\right)}^{2}} \end{array}$$ (3)

Lateral instead of total surface area was chosen to remove the parts that are typically not in contact with airspace in palisade cells.

### Stomatal vaporshed and leaf airspace traits

To extract intercellular airspace (IAS) traits, we first labeled all stomata present in the stacks. Because of the high magnification of our scans, few stomata (*<*10) were generally present, especially in CS shade leaves. This has the potential to bias trait estimates expressed per stoma for stomata near the edges of the stack. To avoid this issue, we only used measurements from stomata that were fully enclosed by other stomata. To label the airspace closest to each stoma, we first computed the whole scan geodesic distance (*L*_geo_), i.e. the shortest path through the airspace from the stomata to any point within the airspace. Then we measured *L*_geo_ for each stoma (*L*_geo,i_) to determine where *L*_geo,i_ equalled *L*_geo_ and thus identify the airspace volume closest to that stoma. This volume closest to one stoma, i.e. the stomatal vaporshed for stoma *i* (Fig. 1), was given a unique value. We then extracted for each stomatal vaporshed the mesophyll cell surface area exposed to the vaporshed (*S*_m,vap_) and the pore volume (*V*_ias,vap_). Using only those stomatal vaporsheds fully enclosed in the scan also allowed for more reliable computation of whole scan median values for geometrical tortuosity (τ) and airspace path lengthening (λ) to estimate the intercellular airspace conductance, *g*_ias_**[see ****Supporting Information—Fig. S3** for additional methods and results**]**.

### Whole-leaf traits

Leaves used for microCT scanning were oven dried at 60°C for 72 h for dry weight measurements. Leaves of 10 additional plants per genotype and treatment were collected, photographed using a digital camera and dried under the same conditions. Images were analyzed using ImageJ to determine total leaf size and leaf mass per area (LMA: leaf dry weight per unit leaf area).

### Biochemical analysis

To test if grapevines adjust their light harvesting capacity to differences in the light environment, chlorophyll concentration was determined in six plants per treatment 3 days before microCT campaigns. From each plant, three different leaves were selected and from each of those three leaf discs (24.1 mm^2^ per disc) were sampled in a 2-mL microtube and immediately snap-frozen in liquid nitrogen. A second set of leaf discs was collected from exactly the same leaves and used to determine the dry weight of three discs. Extraction was performed as described in Herrera *et al.* (2021) by adding 1 mL of dimethylsulfoxide to the sample and shaking it for 15 min under dark conditions and ambient temperature. The extract was then centrifuged for 5 min at 12°C (17 000*g*), and the supernatant placed in a 2-mL microtube. The extraction procedure was repeated twice for each sample, and the combined supernatant was used to measure absorbance in the spectrophotometer (Genesys 10S, Thermo-Scientific). The absorption value was related to concentration as described in Wellburn (1994). Chlorophyll content per volume was computed using the leaf cellular volume (i.e. whole leaf including the epidermis and veins, but excluding airspaces) per area, obtained from the microCT leaf traits analysis above.

### Analysis of trait contribution to *S*_m,LA_

To understand how variation in leaf anatomical traits is affected by underlying components we used a log–log scaling slope analysis (Poorter and van der Werf, 1998; Renton and Poorter, 2011). Thus, for every factor in Eq. (1) one can define a response coefficient (RC) as


$$\begin{array}{l} RC_{x}=\frac{log\left(x_{sun}\right)-log\left(x_{shade}\right)}{log\left(S_{m,LA,sun}\right)-log\left(S_{m,LA,shade}\right)} \end{array}$$ (4)

where *x* is one of *S*_m,Vcl_, 1 − θ_ air_, *f*_mes_ and *L*_leaf_. Combined with Eq. (1) this yields:


$$\begin{array}{l} RC_{S_{m,LA}}=1=RC_{S_{m,Vcl}}+RC_{1-\theta_{air}}+RC_{f_{mes}}+RC_{L_{leaf}} \end{array}$$ (5)

Thus, a change in *S*_m,LA_ can be expressed as a sum of response coefficients, allowing for the expression of each factor as a percentage of the total change. To estimate the overall contribution of the traits, we first log-transformed all values, then computed mean log-transformed values of each trait for each cultivar and light growth condition. We then used these mean values to recompute a mean log-transformed *S*_m,LA_. The difference between log-transformed trait means was then used to calculate the response coefficients in Eq. (4). The results were expressed as the relative contribution of the response coefficient of each of the four traits to the response coefficient for *S*_m,LA_, for which we validated that they summed up to 1 as in Eq. (5).

### Statistical analysis

All statistical analyses were carried out using R 4.1.2 (R Core Team, 2021). Anatomical data were analyzed using mixed effect models (package *nlme*, Pinheiro, 2022), testing single effects of the genotypes and light environment as well as the interaction between the two factors. Individual plants were treated as a random variable in the model. Single effects and interactions were considered significant at *P*-values *<* 0.05. For the gas exchange data, which consisted of one value per plant, an ANOVA was performed using the same single effects and interactions as above. Pearson correlations were computed using the *cor* function of R, and the significance of each correlation was tested using the *cor.mtest* function of the *corrplot* R package (Wei and Simko, 2021).

## Results

### Length, surface, volume and biochemical trait variations between the sun and shade phenotypes.

**Figure 3.**
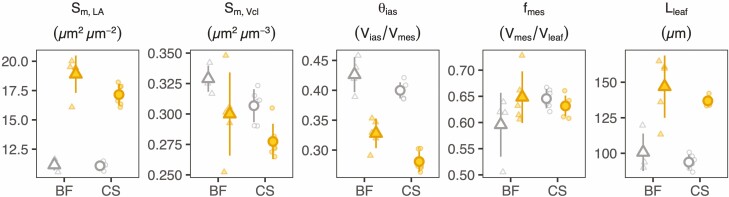
Anatomical measurements made on shade (gray open) and sun (orange filled) leaves of Blaufränkisch (BF, triangles) and Cabernet Sauvignon (CS, circles). Surface area of mesophyll cells exposed to the airspace (*S*_m_) expressed per leaf area (*S*_m,LA_) and per mesophyll cell volume (*S*_m,Vcl_), mesophyll porosity (θ_ ias_), fraction of mesophyll volume within the whole-leaf volume (*f*_mes_) and thickness of the whole leaf (*L*_leaf_). Each symbol represents the mean leaf value taken from two (BF) or three (CS) replicate scans. Five (BF) or six (CS) leaves were imaged per treatment. Large symbols with vertical bars represent means ± one standard deviation and smaller symbols the values for individual leaves. Statistical results are presented in **Supporting Information—Table S1**.

As expected, a higher growth light intensity resulted in significantly larger *S*_m,LA_, but interestingly, a smaller exposed surface area per unit cell volume (*S*_m,Vcl_). We also confirmed that sun leaves were thicker (*L*_leaf_) and substantially less porous (Fig. 3; **see ****Supporting Information—Table S1**).The fraction of mesophyll (airspace and cells) within the leaf volume (*f*_mes_) was not significantly affected by the growth light environment. In addition, stomatal density, vein density and LMA were higher in sun leaves **[see ****Supporting Information—Fig. S2****]**. The LMA was positively and linearly related to leaf cellular volume (see **Supporting Information—Fig. S7**; *P <* 0.0001, *R*^2^ = 0.83, df = 21). High light treatment was linked to a significantly smaller leaf area only in CS leaves **[see ****Supporting Information—Fig. S1**; **Table S1****]**. The total chlorophyll content per unit leaf volume was 45 % (CS) and 25 % (BF) smaller in sun leaves compared to shade leaves (with similar results per unit dry weight; **see ****Supporting Information—Table S2**). However, the ratio between chlorophyll *a* and *b* was only significantly different for CS (sun vs. shade: 3.19 ± 0.02 vs 2.92 ± 0.05, *P <* 0.01 (CS); 2.94 ± 0.11 vs. 2.75 ± 0.03, *P* = 0.12 (BF)).

### Palisade cells have contrasting shape, density and surface-to-volume ratio under sun and shade

**Figure 4.**
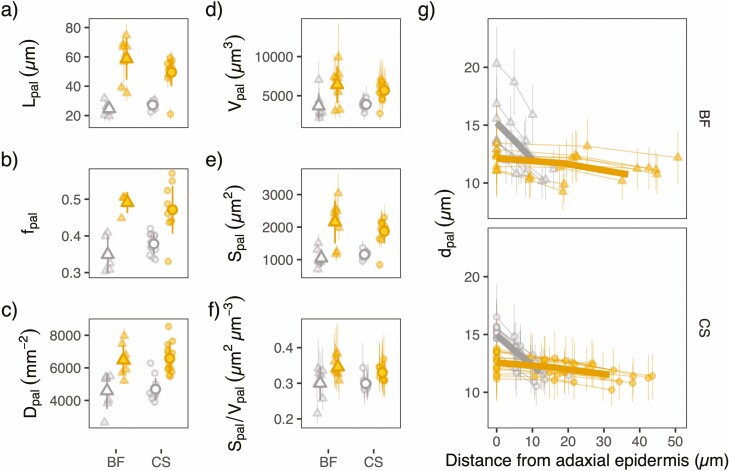
Properties of the palisade mesophyll (a–c) and of the palisade cells (d–g) for shade (gray open) and sun (orange filled) leaves of Blaufränkisch (BF) and Cabernet Sauvignon (CS). Whole palisade traits presented are palisade thickness (a: *L*_pal_), fraction of palisade mesophyll within the mesophyll (b: *f*_pal_), and palisade cell density (c: *D*_pal_). Single cell volume (d: *V*_pal_), surface area (e: *S*_pal_), and their ratio (f: *S*_pal_/*V*_pal_) were computed from median cell diameter measured at the top, middle, and bottom palisade mesophyll in each scan (> 100 cells used to compute the median), where cells were approximated as two truncated cones on top of each other (see Fig. 2). Large symbols with vertical bars represent means of all leaves ± one standard deviation. The means ± one standard deviation for individual leaves are indicated by smaller symbols with vertical bars in a, d, e, f and g, and for individual scans (multiple scans per leaf) in b and c.

Sun leaves had thicker palisade tissue which was associated with more elongated cells (Figs. 1 and 4; **[see ****Supporting Information—Table S1****]**) with a smaller cell diameter. In addition, a difference in cell shape was observed: while sun leaves palisade cells were shaped capsule-like, shade leaves had more funnel-shaped palisade cells (difference of slope between sun and shade leaves in Fig. 4g), with the minimum diameter being about the same between sun and shade leaves, but the diameter near the adaxial epidermis being substantially smaller in sun leaves (~25 %). This allowed for a greater palisade cell density in sun leaves (Fig. 4c). Despite the smaller basal diameter of sun-leaf palisade cells in comparison to shade leaves individual cells in the palisade tissue of sun leaves had a larger volume (*V*_pal_), lateral surface (*S*_pal_) and *S*_pal_/*V*_pal_ ratio **[see ****Supporting Information—Table S1****]**.

### More surface area per airspace volume in sun leaf vaporsheds

**Figure 5.**
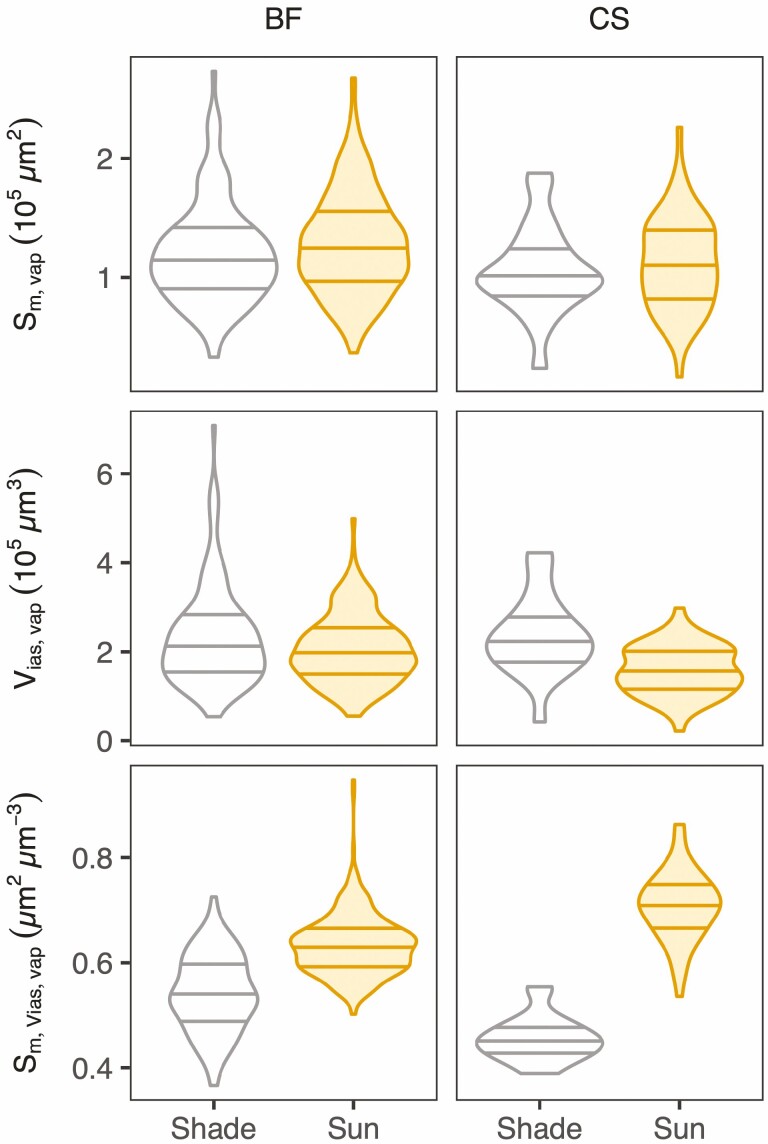
Surface area of mesophyll cells exposed to airspace (*S*_m,vap_) and volume of airspace (*V*_ias,vap_) within stomatal vaporsheds, and the surface to volume ratio (*S*_m,Vias,vap_) for shade (open) and sun (filled) leaves of Blaufränkisch (BF) and Cabernet Sauvignon (CS). Lines within the violins represent the first quartile (top), median (middle), and third quartile (bottom) for all analyzed vaporsheds (*n* = 55 (CS-sun), 21 (CS-shade), 317 (BF-sun) and 122 (BF-shade)). Five (BF) to six (CS) leaves were imaged per treatment. Statistics are presented in **Supporting Information—Table S1**.

For both cultivars, the mesophyll surface area per vaporshed volume was similar between sun and shade leaves (*S*_m,vap_; Fig. 5; **see ****Supporting Information—Table S1**). However, airspace volume per vaporshed was significantly lower in sun leaves. This led to vaporsheds of sun leaves having larger *S*_m,Vias,vap_, and this effect was substantially and significantly stronger in CS than in BF (similar for *S*_m,Vias_ at the whole-leaf level; **see ****Supporting Information—Fig. S1**). *S*_m,Vias_ ratio at the whole leaf level is a good predictor of the amount of surface available for diffusion on a leaf area basis (i.e. *S*_m,LA_; **see ****Supporting Information—Fig. S1**). Out of the airspace geometrical traits, only path lengthening became significantly longer under high-light growth conditions, while airspace tortuosity was not affected **[see ****Supporting Information—Fig. S3**; **Table S1****]**.

### Contribution of anatomical traits to sun-induced differences in *S*_m,LA_

**Figure 6.**
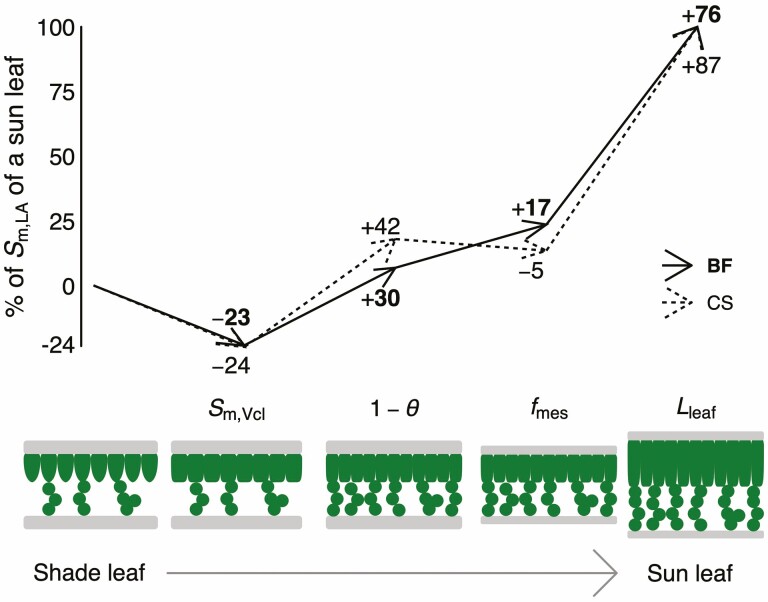
Relative contribution of *S*_m,Vcl_, θ_ ias_, *f*_mes_ and *L*_leaf_ to the variation of *S*_m,LA_ from shade (left) to sun leaves (right) for Blaufränkisch (BF; solid lines and bold font) and Cabernet Sauvignon (CS; dashed lines and normal font). Upward arrows indicate more *S*_m,LA_ from shade to sun, while downward arrows indicate a reduction in *S*_m,LA_, mainly observed for *S*_m,Vcl_. The scale to the left shows the cumulative contribution of each trait to the difference in *S*_m,LA_ observed between shade and sun leaves (i.e. 100 % corresponds to a sun leaf), while the numbers along the arrows indicate the relative contribution of each trait for each cultivar. The cartoons represent a simplified visualization of the effect contributed by each individual anatomical trait (*S*_m,Vcl_, θ_ ias_, *f*_mes_ and *L*_leaf_).

High growth light intensity was associated with larger *S*_m_ when expressed per unit leaf area (*S*_m,LA_; Fig. 3; **Supporting Information—Table S1**). Of all the four traits that contribute to variations in mean *S*_m,LA_, leaf thickness had the most substantial impact, with the greater thickness in sun leaves being responsible for 76 % (BF) and 87 % (CS) of the difference in *S*_m,LA_ (Fig. 6). The lower porosity was the second largest component, contributing to 30 % (BF) and 42 % (CS) of the difference in *S*_m,LA_. Variations in the fraction of mesophyll in the leaf also had a considerable effect on *S*_m,LA_ in BF (17 %), but had a minor contribution in CS (−5 %). Summing the effects of these components leads to a larger (*>*100 %) difference in *S*_m,LA_ than what was actually observed. This can be explained by the effect of the cell surface to volume ratio, *S*_m,Vcl_, which was significantly smaller under high light (Fig. 3; **[see ****Supporting Information—Table S1****]**) and thus substantially lowered *S*_m,LA_ (by 23 % in BF and 24 % in CS).

### Photosynthesis expressed per leaf area, mass and volume

**Figure 7.**
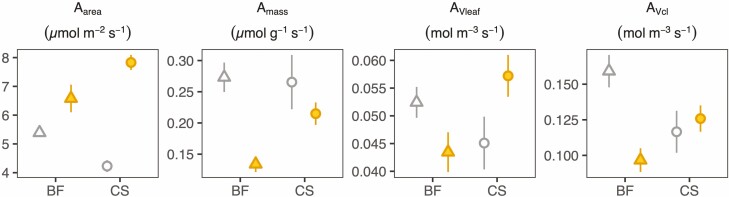
Photosynthesis measured at 1000 µmol m^−2^ s^−1^ expressed per unit leaf area (*A*_area_), per unit leaf dry mass (*A*_mass_), per unit leaf volume (*A*_Vleaf_) and per unit mesophyll cell volume (*A*_Vcl_) for shade (open) and sun (filled) leaves of Blaufränkisch (BF) and Cabernet Sauvignon (CS). Points present the mean values, and vertical lines present one standard error. *n* = 24 (CS-sun), 9 (CS-shade), 4 (BF-sun) and 8 (BF-shade). Statistics are presented in **Supporting Information—Table S1**.

Higher area-based photosynthesis (*A*_area_) at close to light-saturated conditions (i.e. 1000 µmol m^−2^s^−1^) was observed in sun grown leaves of both genotypes (Fig. 7), but when it was integrated over the whole leaf area it was not different between sun and shade CS leaves (*A*_leaf_; *P*-values of Tukey-HSD pairwise comparison between sun and shade: 0.99 (CS) and *<*0.0001 (BF); **Supporting Information—Table S1**). When expressed per unit mass (*A*_mass_), the rates were lower in sun leaves of both cultivars (Fig. 7). On a leaf volume basis (*A*_Vleaf_), the differences were small for CS and non-significant for BF **[see ****Supporting Information—Table S1****]**. However, as mesophyll cell volume per leaf area in sun leaves was larger than in shade leaves, expressing photosynthesis on a mesophyll cell volume basis (*A*_Vcells_) shows a full overlap for both growth light environments for CS, while for BF sun leaves assimilate less per mesophyll cell volume. These results parallel those observed for *A*_mass_.

## Discussion

Contrasting growth light intensity lead to marked anatomical and physiological differences in grapevine leaves, with sun leaves having both faster assimilation rates and more dry mass per unit area, similar to previous studies using comparable differences in light levels (Schultz and Matthews, 1993; Vanden Heuvel *et al.*, 2004; González *et al.*, 2021). In agreement with existing work on grapevine and other species (Chavarria *et al.*, 2012; Poorter *et al.*, 2019; González *et al.*, 2021), chlorophyll content per unit mass was lower in sun leaves, but was not affected much when expressed per unit leaf area **[see ****Supporting Information—Table S2****]**. This suggests that sun and shade leaves can absorb similar amounts of light energy per unit area. Therefore, the observed higher area-based photosynthetic activity in sun leaves is most likely resulting from greater electron transport and/or Rubisco capacity per unit area (Evans and Poorter, 2001).

Photosynthesis is not only limited by biochemical capacity, but also by the CO_2_ diffusion resistance through the stomata and the mesophyll (Oguchi *et al.*, 2018). The mesophyll diffusion resistance can be divided into a resistance through intercellular air spaces and through the liquid phase (including cell walls, membranes, cytoplasm and chloroplast stroma). Even when considering the greater stomatal density, thicker and denser leaves would typically result in a longer diffusion path length (Earles *et. al*, 2018; Harwood *et al.*, 2021). As a result, thinner or more porous leaves might benefit photosynthesis in sun leaves, because airspace resistance would be reduced (see Eq. (8) at **Supporting Information—Fig. S3**; Oguchi *et al.*, 2018). However, sun leaves are typically thicker and less porous than shade leaves (Fig. 3, also Poorter *et al.*, 2019, 2022). The resistance through airspaces is typically only a minor part of the total diffusion resistance and the increase in mesophyll surface area available for diffusion across the liquid phase outweighs the effect of a longer path length: we estimated that although *g*_ias_ in sun leaves may be up to 40 % smaller, the larger *S*_m,LA_ would nearly double the liquid phase part of the mesophyll conductance (*g*_liq_; **see ****Supporting Information—Fig. S4**).

We observed the expected larger *S*_m,LA_ for sun leaves of both genotypes, and determined how this anatomical feature could be decomposed into other anatomical traits. It is typically assumed that leaves increase their lamina thickness to allow for a larger *S*_m,LA_ (Terashima *et al.*, 2001). The mesophyll and both upper and lower epidermis were thicker in sun leaves, though the differences in the epidermises were less pronounced, in agreement with previous studies on grapevine (Palliotti *et al.*, 2000 for all tissues; González *et al.*, 2021 for whole leaf thickness). We confirmed that thickness was a major contributor to the larger *S*_m,LA_ in sun leaves, but showed that lower leaf porosity also contributes significantly. This is supported by recent results showing that variation in cell-to-cell contact, cell shape and porosity lead to large variations in *S*_m,LA_ within *Eucalyptus* species of similar mesophyll thickness (Harwood *et al.*, 2021). Sun leaves would also benefit from a larger *S*_m,Vcl_, which could be achieved by smaller cells, less connectivity between cells, or an altered cell shape, such as more lobing. We found that the volume of individual palisade cells was larger in sun leaves (Fig. 4d). This bigger volume was mainly the result of longer palisade cells. Surprisingly, despite being bigger, these cells had a slightly larger lateral surface-to-volume ratio, related to a shift from a more funnel-like shape in the shade to a cylindrical shape in the sun leaves (Fig. 4g). Similar shape differences have been reported in the past (Turrell, 1936; Chabot and Chabot, 1977; Lee *et al.*, 1990). Ideally shaped cylinders have a larger surface-to-volume ratio compared to truncated cones. However, a more cylindrical shape allows for a closer packing of the cells in the tissue (evident from the decreased porosity), which effectively decreased the amount of exposed surface area (8–11 % less *S*_m,Vcl_ at the whole leaf level, *P* = 0.004; **Supporting Information—Table S1**). Terashima *et al.* (2001) previously argued that having smaller cells would be beneficial to sun leaves, but that constraints related to leaf development or increased nitrogen cost for genetic material may prevent this. Moreover, it has been argued that a reduction in genome size (and therefore N and P cost per cell) enabled angiosperm lineages to reduce mesophyll cell size (Théroux-Rancourt *et al.*, 2021). The explanation for the larger volume of palisade cells in sun leaves may therefore be that cell expansion offers a cheap way to construct thicker, denser leaves, and thus allows for a large *S*_m,LA_ under high light.

The model presented in Eq. (1) describes how different aspects of the anatomy interact and influence *S*_m,LA_. It is worth noting that leaf anatomy may be under multiple selective pressures and that the function of traits cannot be completely understood when only looking at their relevance for gas-exchange. For example, the higher airspace fraction in thin shade leaves would allow them to maintain bending stiffness (Onoda *et al.* 2008). Moreover, the dependency between the leaf traits in Eq. (1) says nothing about which traits are under selective pressure (as discussed by Chatelet *et al.*, 2013) and which are under genetic control (Lehmeier *et al.*, 2017; Lundgren *et al.*, 2019; Lundgren and Fleming, 2020). Discussion about this topic would benefit from a clear description of the intrinsic relation between these traits. A similar model to the one presented in Eq. (1) can be formulated for the relationship between LMA and *S*_m,LA_**[see ****Supporting Information—Fig. S6****]**, which may help elucidate the mechanistic basis of the relationship between LMA and mesophyll conductance (Muir *et al.*, 2014).

### Differences in construction strategies

Photosynthesis per unit dry mass was lower in sun leaves, although the difference was much more pronounced in BF (~52 %) compared to CS (~19 %). When expressed per unit mesophyll cell volume, a lower photosynthesis rate was only observed in BF sun leaves, despite more mass per unit volume investment. These findings suggest that in sun leaves of BF, more mass is allocated to support structures (e.g. cell walls). Such an increase in support structures may allow for larger leaves, and indeed this was observed, with leaf area in BF being similar between sun and shade, while being smaller in sun leaves for CS **[see ****Supporting Information—Fig. S1****]**. A smaller leaf area under high light has also been found by (Cartechini and Palliotti, 1995; Palliotti *et al.*, 2000; González *et al.*, 2021), while others reported constant or similar leaf area (Schultz and Matthews, 1993), suggesting that the dose-response curve of growth irradiance on leaf area is dependent on growth conditions or the genotype under investigation (Vanden Heuvel *et al.*, 2004). Given that CS and BF are genetically distant from each other (Magris *et al.*, 2021), different construction strategies in response to the light environment could explain the contrasting responses. However, since our two cultivars were grown at different times during the year (spring vs summer) and on slightly different soils, we cannot exclude an effect of the growth conditions. We have observed variations in the anatomical responses to shading in several genotypes in an experimental vineyard. In the vineyard, 5-year-old CS plants had similar differences between sun and shade leaves as were found in the greenhouse experiment (**see ****Supporting Information—Fig. S8**; using the same shading cloth reducing PPFD by 60 %). More research is needed in vineyard settings to understand the relevance of such different strategies.

### Stomatal vaporsheds as mesophyll building blocks for the facilitation of carbon assimilation

We introduced a new anatomical unit, the stomatal vaporshed, that can be seen as a building block for the construction of the mesophyll. The stomata-to-diffusive-surface pathway (Fig. 1) is a key aspect of mesophyll structure. Here, we described this pathway in detail and showed that there is substantial anatomical variation between varporsheds (Fig. 5; see also Borsuk *et al.* (2022) for an anatomical description of the spongy mesophyll air channels and description of similar anatomical variation). Yet, if we average over a leaf section of substantially larger volume, the median vaporshed values match the values computed on whole scans **[see ****Supporting Information—Table S1****]**. We consider part of the work presented here needed to assess the validity of bulk leaf averages, or more precisely ‘bulk scan’ averages.

Our analysis of airspace traits provides a basis for the commonly used dimensional abstraction when moving from an inherently 3D leaf structure to a 1D resistance model. After entering the stomatal pore, the CO_2_ flux needs to spread out over an ever increasing intracellular air volume, before dissolving into apoplastic water and diffusing towards the chloroplasts. Pickard (1981) described this using a model with concentric hemispherical domains (the stomatal cavity, the porous mesophyll) that roughly maps onto our stomatal vaporshed. The vaporshed thus becomes a useful unit for numerical models analyzing CO_2_ diffusion in a realistic leaf structure (Ho *et al*. 2016). The data made available here will foster further research in that direction.

## Conclusions and Practical Implications

Two-dimentional growth, i.e. growth of the planal leaf area, is required to increase light capture (Roderick *et al.*, 1999; Moulia *et al.*, 2021). At the same time, photosynthetic capacity typically scales with leaf volume (Roderick *et al.*, 1999), but is still limited by diffusion over the internal surface area of the mesophyll cells. Our results indicate that low porosity and thick leaves are key structural alterations needed to maximize carbon assimilation under high light conditions. Despite slightly greater airspace diffusion resistance, both leaf thickness and porosity positively affected the amount of mesophyll surface area available for CO_2_ diffusion per unit leaf area. Moreover, we showed that a change in palisade mesophyll cell shape, a trait available through our 3D image analysis, led to slightly less cell-to-cell contact, which negatively affected the surface area available for diffusion. However, since the development of thicker leaves by more elongated palisade cells likely requires less resources than by increasing cell numbers (Terashima *et al.*, 2001), this represents a cost-efficient way to achieve high rates of photosynthesis in sun leaves. The disentangling of the effects of different traits involved in building the total internal surface area available for diffusion in leaves was made possible using a method that explicitly describes their interdependence. Such an analysis is necessary to clarify how characteristics changed by growth and development affect functional leaf traits.

Shading in vineyards happens due to multiple causes: natural shade due to training systems, shoot positioning, or dense canopies (leaves shading leaves), but also through the use of protection nets (e.g. against hail or birds) or shading nets against excessive heat stress. Depending on the growth conditions or genotypes under consideration, different strategies may exist to maintain whole-plant carbon balance under contrasting light availability. Under the growth conditions of the CS plants investigated here, individual shade leaves acquired as much CO_2_ per leaf as sun leaves. By contrast, photosynthetic rate per leaf in shaded BF plants was about half of that of sun leaves. Thus, while shade leaves can provide a substantial contribution to total carbon balance for certain combinations of genotypes and growth conditions, they may contribute little for other combinations. Hence, shading management could benefit from genotype-specific approaches.

## Supporting Information

The following additional information is available in the online version of this article—

**Table S1.** *P*-values for the statistical analysis of the different trait responses.

**Table S2.** Chlorophyll *a*, *b*, and *a*+*b* (total) concentration per dry weigth, leaf area, and leaf volume in leaf samples grown under sun or shade conditions from the Cabernet Sauvignon (CS) and Blaufränkisch (BF) experiments.

**Figure S1.** Thickness (µm) of the abaxial epidermis (*L*_ep,ab_), adaxial epidermis (*L*_ep,ad_), and of the mesophyll (*L*_mes_); leaf mass per area (LMA); leaf area (LA); the exposed surface area of mesophyll cells per whole mesophyll volume (*S*_m,Vcl+ias_), per leaf volume between epidermes (*S*_m,Vleaf-ep_), and per airspace volume (*S*_m,Vias_); and stomatal (*D*_stom_) and vein densities (*D*_V_) for shade and sun leaves of Blaufränkisch (BF) and Cabernet Sauvignon (CS).

**Figure S2.** Fraction of adaxial and abaxial epidermes (*f*_ep,ad_ and *f*_ep,ab_), mesophyll cells (*f*_cells_), airspace (*f*_ias_), and vascular tissue (*f*_vasc_) over the total leaf volume for shade and sun leaves of Blaufränkisch (BF) and Cabernet Sauvignon (CS).

**Figure S3.** Path lengthening λ and tortuosity τ of the airspace, computed from the stomatal pores to the mesophyll cell surfaces for shade and sun leaves of Blaufränkisch (BF) and Cabernet Sauvignon (CS).

**Figure S4.** Estimated values of the airspace (*g*_ias_) and liquid phase (*g*_liq_) components of mesophyll conductance, showing that *g*_ias_ is about ten times higher than *g*_liq_, indicating that the airspace imposes a much lower resistance to gas diffusion than the cell wall and intracellular components.

**Figure S5.** Correlation between airspace tortuosity, path lengthening, and surface area traits.

**Figure S6.** Contribution of individual traits to the difference in LMA between shade and sun leaves in Blaufränkisch (BF) and Cabernet Sauvignon (CS).

**Figure S7.** Relationship between leaf mass per area (LMA) and the extracted leaf cellular volume (total leaf volume minus airspace volume) or the mesophyll cell volume.

**Figure S8.** Anatomical values for vineyard-grown grapevine cultivars (5 years old plants) for shade (60 % light transmission reduction) and sun grown leaves.

plad001_suppl_Supplementary_Material

## Data Availability

All imaging data (raw microCT scans and segmented scans) and data extracted from those images are available on Zenodo (https://doi.org/10.5281/zenodo.5994663). Code created to extract stomatal vaporsheds is included in the *plant-leaf-microct* suite, within the *leaf-traits-microct* program (Théroux-Rancourt *et al.*, 2020a, https://github.com/plant-microct-tools/leaf-traits-microct).
