# Supplementary material for: Analyzing anatomy over three dimensions unpacks the differences in mesophyll diffusive area between sun and shade Vitis vinifera leaves
Source: AoB Plants. 2023 Jan 25;15(2):plad001. doi: 10.1093/aobpla/plad001 (PMC10029806; doi:10.1093/aobpla/plad001)
Supplement: plad001_suppl_Supplementary_Material [file plad001_suppl_supplementary_material.pdf]

Analyzing anatomy over three dimensions unpacks the differences in mesophyll diffusive area between sun and shade *Vitis vinifera* leaves

Guillaume Théroux-Rancourt[1*], José Carlos Herrera[2], Klara Voggeneder[1], Federica De Berardinis[2], Natascha Luijken[1], Laura Nocker[2], Tadeja Savi[1], Susanne Scheffknecht[1], Moritz Schneck[1], Danny Tholen[1]

[1] University of Natural Resources and Life Sciences, Vienna, Department of Integrative Biology and Biodiversity Research, Institute of Botany, 1180 Vienna, Austria

[2] University of Natural Resources and Life Sciences, Vienna, Department of Crop Sciences, Institute of Viticulture and Pomology, 3430 Tulln an der Donau, Austria

[*]corresponding author, guillaume.theroux-rancourt@boku.ac.at

Table S1: p-values for the statistical analysis of the difference trait ressponses. Degrees of freedom (df) with two values represent numerator and denominator df values in mixed model analyses. CV refers to the effect of the Blaufränkisch (BF) and Cabernet Sauvignon (CS) experiments, and Light refers to the sun or shade treatment.

variable	definition	units	df	p-values		
				CV	Light	CV:Light
**Thickness**						
$L_{ep,ab}$	Abaxial epidermis thickness	µm	17 / 32	0.3202	0.0001	0.1257
$L_{ep,ad}$	Adaxial epidermis thickness	µm	17 / 32	0.0079	0.0092	0.8930
L_{leaf}	Leaf thickness	µm	17 / 32	0.0817	< 0.0001	0.8069
L_{mes}	Mesophyll thickness	µm	17 / 32	0.1343	< 0.0001	0.6253
L_{pal}	Palisade thickness	µm	28	0.1666	< 0.0001	0.0792
**Other anatomy**						
LMA	Leaf mass per area	$g\ m^{-2}$	20	0.0023	< 0.0001	0.0748
LA	Leaf area	cm^2	20	< 0.0001	0.4591	0.0001
θ_{ias}	Mesophyll porosity	$\mu m^3\ \mu m^{-3}$	17 / 32	0.0007	< 0.0001	0.1793
**Exposed surface area ratios**						
$S_{m,LA}$	S_m per leaf area	$m^2\ m^{-2}$	17 / 32	0.0222	< 0.0001	0.0970
$S_{m,Vcl}$	S_m per mesophyll cell volume	$\mu m^2\ \mu m^{-3}$	17 / 32	0.0097	0.0039	0.7389
$S_{m,Vias}$	S_m per airspace volume	$\mu m^2\ \mu m^{-3}$	17 / 32	0.0143	< 0.0001	0.0298
**Fraction of tissue per leaf volume**						
f_{mes}	Mesophyll (cells+airspace)	$\mu m^3\ \mu m^{-3}$	17 / 32	0.4198	0.0876	0.2309
f_{cells}	Mesophyll cells	$\mu m^3\ \mu m^{-3}$	17 / 32	0.0030	< 0.0001	0.5479
f_{ias}	Airspace	$\mu m^3\ \mu m^{-3}$	17 / 32	0.0702	< 0.0001	0.0234
$f_{ep,ab}$	Abaxial epidermis	$\mu m^3\ \mu m^{-3}$	17 / 32	0.8880	0.0031	0.1067
$f_{ep,ad}$	Adaxial epidermis	$\mu m^3\ \mu m^{-3}$	17 / 32	0.0637	< 0.0001	0.6692
f_{vasc}	Vascular	$\mu m^3\ \mu m^{-3}$	17 / 32	0.2865	0.4350	0.7908
**Light saturated photosynthesis**						
A_{Vleaf}	per leaf volume	$mol\ m^{-3}\ s^{-1}$	19	0.1688	0.0796	0.1875
A_{Vcl}	per mesophyll cell volume	$mol\ m^{-3}\ s^{-1}$	19	0.8293	0.3427	0.0297
A_{Vias}	per airspace volume	$mol\ m^{-3}\ s^{-1}$	19	0.0023	< 0.0001	0.0015
A_{mass}	per leaf dry mass	$\mu mol\ g^{-1}\ s^{-1}$	19	0.0025	0.0003	0.1570
A_{area}	per area	$\mu mol\ m^{-2}\ s^{-1}$	19	0.3358	< 0.0001	0.1570
A_{leaf}	per whole leaf	$\mu mol\ leaf^{-1}\ s^{-1}$	19	< 0.0001	0.0001	0.0002
**Intercellular airspace traits**						
λ	diffusive path lengthening	$m\ m^{-1}$	17 / 40	0.2149	0.0038	0.4805
τ	airspace tortuosity factor	$m^2\ m^{-2}$	17 / 40	0.0001	0.1781	0.3558
$\tau \times \lambda$			17 / 40	0.0945	0.0172	0.3798
g_{ias}	Airspace conductance	$mol\ m^{-2}\ s^{-1}$	17 / 40	0.9720	0.0009	0.5454
**Stomatal vaporsheds**						
$S_{m,vap}$	Exposed SA per vaporshed	μm^2	17 / 494	0.1596	0.9197	0.6992
$V_{ias,vap}$	Airspace volume per vaporshed	μm^3	17 / 494	0.2771	0.0128	0.7023
$S_{m,Vias,vap}$	Airspace SA/V per vaporshed	$\mu m^2\ \mu m^{-3}$	17 / 494	0.4888	< 0.0001	0.0018
**Palisade mesophyll traits**						
V_{pal}	Mean palisade cell volume	μm^3	28	0.4653	0.0001	0.3702
S_{pal}	Mean palisade cell lateral SA	μm^2	28	0.3462	< 0.0001	0.1636
S/V_{pal}	Mean palisade cell SA/V	$\mu m^2\ \mu m^{-3}$	28	0.4388	0.0004	0.5636
D_{pal}	Palisade cell packing density	mm^{-2}	28	0.7334	< 0.0001	0.7963
f_{pal}	Fraction of palisade per mesophyll thickness	$\mu m\ \mu m^{-1}$	16	0.8608	0.0001	0.3834

Table S2: Chlorophyll a, b, and a+b (total) concentration per dry weigth, leaf area, and leaf volume in leaf samples grown under sun or shade conditions from the Cabernet Sauvignon (CS) and Blaufränkisch (BF) experiments. p-values for a two way ANOVA written below each chlorophyll type.

CV	Treatment	mg g^{-1}	µg mm^{-2}	µg mm^{-3}
**Chlorophyll a**				
BF	Sun	2.47±0.14	0.235±0.011	1.60±0.08
	Shade	3.39±0.22	0.214±0.011	2.12±0.11
CS	Sun	3.32±0.37	0.132±0.016	0.96±0.11
	Shade	9.12±0.46	0.159±0.015	1.70±0.16
p-values	CV	< 0.0001	< 0.0001	0.0002
	Light	< 0.0001	0.9567	< 0.0001
	CV:Light	< 0.0001	0.0791	0.3686
**Chlorophyll b**				
BF	Sun	0.84±0.04	0.081±0.005	0.55±0.03
	Shade	1.23±0.08	0.078±0.004	0.77±0.04
CS	Sun	1.04±0.12	0.041±0.005	0.30±0.03
	Shade	3.12±0.11	0.054±0.004	0.58±0.05
p-values	CV	< 0.0001	< 0.0001	< 0.0001
	Light	< 0.0001	0.3426	< 0.0001
	CV:Light	< 0.0001	0.0972	0.4851
**Total**				
BF	Sun	3.30±0.17	0.316±0.015	2.15±0.10
	Shade	4.63±0.30	0.292±0.015	2.89±0.15
CS	Sun	4.36±0.49	0.173±0.020	1.26±0.15
	Shade	12.25±0.57	0.214±0.019	2.28±0.21
p-values	CV	< 0.0001	< 0.0001	0.0001
	Light	< 0.0001	0.7691	< 0.0001
	CV:Light	< 0.0001	0.0779	0.3897

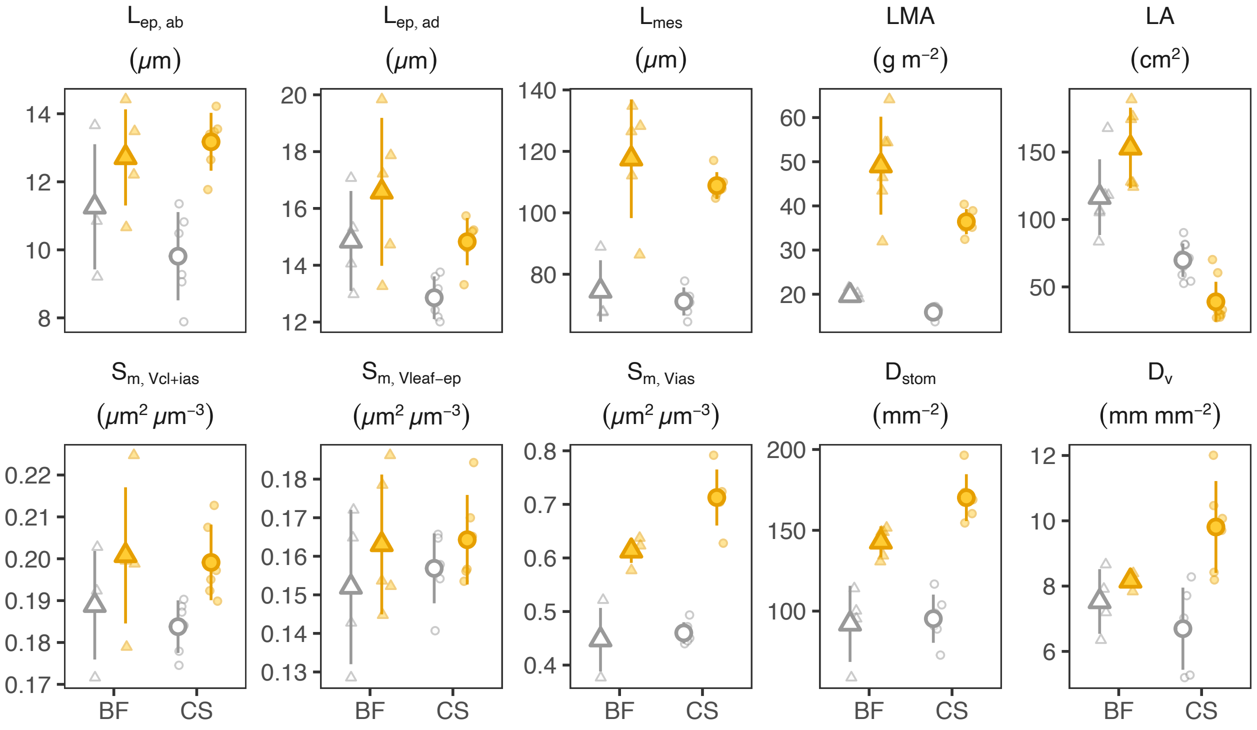

Figure S1: Thickness (μm) of the abaxial epidermis ($L_{ep,ab}$), adaxial epidermis ($L_{ep,ad}$), and of the mesophyll (L_{mes}); leaf mass per area (LMA); leaf area (LA); the exposed surface area of mesophyll cells per whole mesophyll volume ($S_{m„Vcl+ias}$), per leaf volume between epidermes ($S_{m,Vleaf\text{-}ep}$), and per airspace volume ($S_{m,Vias}$); and stomatal (D_{stom}) and vein densities (D_V) for shade (open) and sun (filled) leaves of Blaufränkisch (BF) and Cabernet Sauvignon (CS). Stomata were counted and vein length measured on paradermal views and divided by the paradermal area of the scans to compute D_{stom} and D_V). Each point represents the mean leaf value taken from two (BF) or three (CS) replicate scans. Five (BF) to six (CS) leaves were imaged per treatment. Large symbols with vertical bars represent means ± one standard deviations and smaller symbols the values for individual leaves. Statistical results are presented in Table S1.

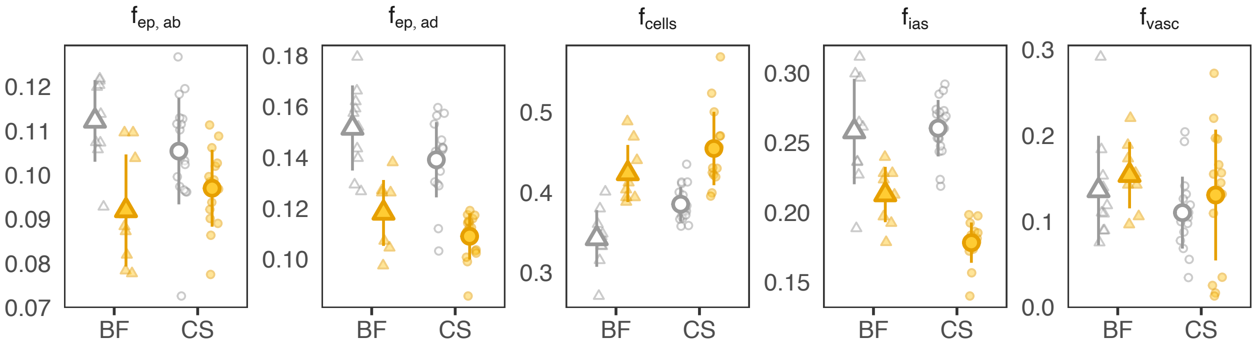

Figure S2: Fraction of adaxial and abaxial epidermes ($f_{ep,ad}$ and $f_{ep,ab}$), mesophyll cells (f_{cells}), airspace (f_{ias}), and vascular tissue (f_{vasc}) over the total leaf volume for shade (gray open) and sun (orange filled) leaves of Blaufränkisch (BF) and Cabernet Sauvignon (CS). Five (BF) to six (CS) leaves were imaged per treatment. Large symbols with vertical bars represent means ± one standard deviations and smaller symbols the individual fractions in each of the two (BF) or three (CS) replicate scans per leaf. Statistical results are presented in Table S1.

Geometrical airspace traits and airspace and liquid-phase conductances: supplementary methods and results

Methods

We used the methods of Earles *et al.* (2018) and implemented them in Python to extract geometrical tortuosity (τ) and airspace path lengthening (λ), both of which use the geometrical distance from the stomata to any point within the leaf. τ and λ are geometrical measures:

$$\tau = \frac{L_{Euc}}{L_{epi}} \tag{6}$$

$$\lambda = \frac{L_{geo}^2}{L_{Euc}^2} \tag{7}$$

where L_{Euc} is the Euclidean distance, i.e. the unobstructed distance from stomata to any point within the airspace; L_{epi} the unobstructed distance from the epidermis containing stomata to any point within the airspace; and L_{geo} the geodesic distance, i.e. the shortest path from the stomata to any point within the airspace and accounting for cells. Whole scan estimates were computed for values at the cell surface only within fully enclosed stomatal vaporsheds.

The intercellular airspace conductance, g_{ias}, is then computed using a modified version of the equation of Earles *et al.* (2018):

$$g_{ias} = \frac{\theta_{ias} D_m}{L_{0.5S} \tau \lambda} \tag{8}$$

where θ_{ias} is IAS porosity of the mesophyll, D_m is the diffusivity of CO_2 in air (1.51×10^{-5} m^{-2} s^{-1} at 25 °C), and $L_{0.5S}$ is the distance from the stomata-containing epidermis where 50% of the absorptive surface area is found (close to half of the mesophyll thickness), computed by doing a cumulative summation of surface area along the leaf profile and finding

$L_{0.5S}$, τ the geometrical tortuosity, and λ the airspace path lengthening (see supplementary methods at Figure S3). All values in this equation are from enclosed stomatal vaporsheds only. Liquid-phase conductance (g_{liq}) was computed as a function of $S_{m,LA}$ as in Théroux-Rancourt *et al.* (2021) using the same resistance values for both light environments and a chloroplast coverage of the cell surface of 90%.

Results

The geometrical airspace traits (Figure S3) showed that path lengthening was smaller in sun leaves and that tortuosity was not significantly different between light environments, but that their combined effect on the diffusion pathway ($\tau \times \lambda$) was dominated by the effect of path lengthening, showing only a significant effect of light environment (Table S1; also tied to a highly significant correlation to leaf and mesophyll thickness, e.g. for L_{mes}: df=56, ρ=-0.63, $P < 0.0001$). Moreover, tortuosity was significantly higher in CS than BF. Notwithstanding the lower $\tau \times \lambda$ in sun leaves, the greater mesophyll thickness led to a substantial and significant reduction in g_{ias} expressed per leaf area or mesophyll volume (Figure S4). The combined diffusion pathway ($\tau \times \lambda$) correlated positively with θ_{ias} and inversely with $S_{m,LA}$ and $S_{m,Vias}$ (Figure S5). While g_{ias} did change with growth light environment, it was about one order of magnitude higher than g_{liq} (Figure S4), and thus would contribute less to potential variations in total mesophyll conductance. Absolute values for g_{ias} and g_{liq} may differ from our estimates, but the difference between the conductances is likely to be conserved.

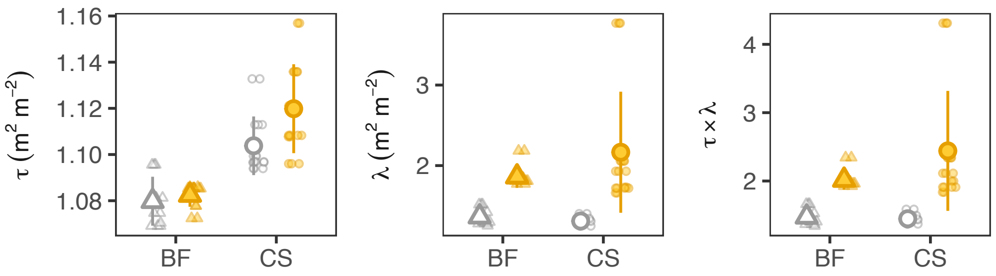

Figure S3: Path lengthening λ and tortuosity τ of the airspace, computed from the stomatal pores to the mesophyll cell surfaces for shade (gray open) and sun (orange filled) leaves of Blaufränkisch (BF) and Cabernet Sauvignon (CS). The rightmost panel is the product of λ and τ as used in Eq. (3). Each point represents the mean leaf value taken from two (BF) or three (CS) replicate scans. Large symbols with vertical bars represent means ± one standard deviations and smaller symbols the values for individual leaves. Five (BF) to six (CS) leaves were imaged per treatment. Statistical results are presented in Table S1.

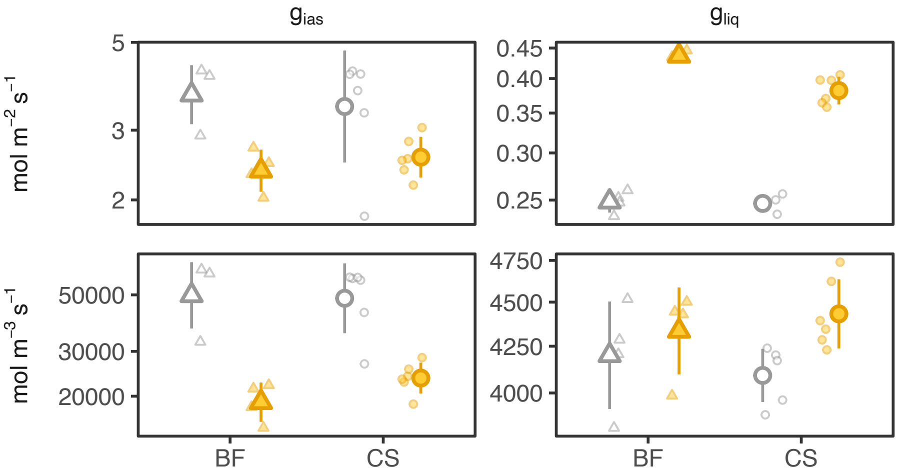

Figure S4: Estimated values of the airspace (g_{ias}) and liquid phase (g_{liq}) components of mesophyll conductance, showing that g_{ias} is about ten times higher than g_{liq}, indicating that the airspace imposes a much lower resistance to gas diffusion than the cell wall and intracellular components. Airspace conductance was estimated using Eq. (8) and g_{liq} was estimated as a function of $S_{m,LA}$ or $S_{m,,Vcl+ias}$ as in Théroux-Rancourt *et al.* (2021) using the same resistance values for both light environments and a chloroplast coverage of the cell surface of 90%. Large symbols with vertical bars represent means ± one standard deviations and small symbols the values for individual leaves

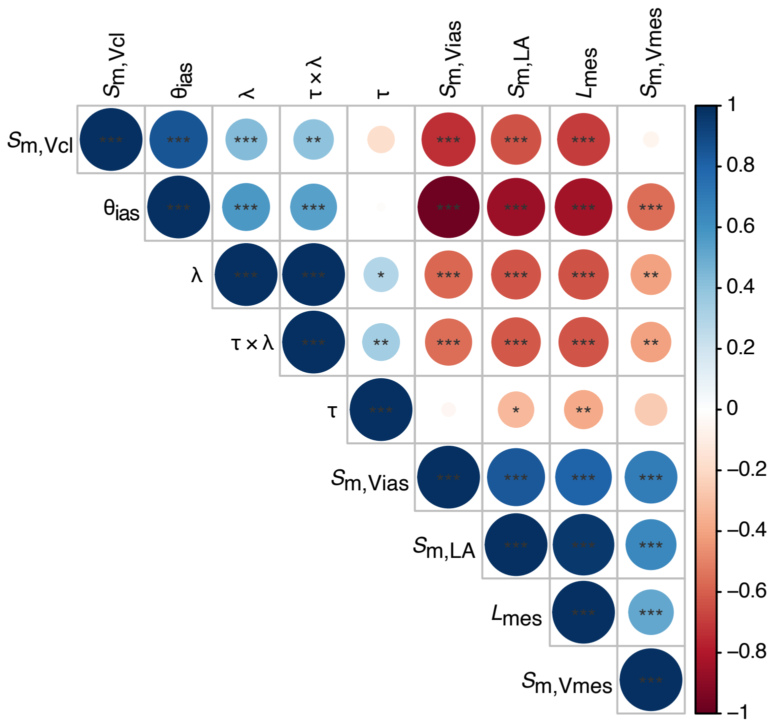

Figure S5: Correlation between airspace tortuosity, path lengthening, and surface area traits. Top shows points sized to the Pearson correlation coefficient value between the two variables, and asterisks indicate the *p*-value of the correlations (*** < 0.001, ** < 0.01, * < 0.05).

Contributions of traits to $S_{m,LA}$ and LMA: supplemental methods and results

Methods

The leaf area of a flat leaf can be expressed in terms of thickness and volume. As a result, $S_{m,LA}$ can be expanded to:

$$S_{m,LA} = S_m \frac{L_{leaf}}{V_{leaf}} \quad (9)$$

The mesophyll cell volume depends on the fraction of mesophyll (including airspaces) in the leaf volume and the fraction of airspaces in the mesophyll volume:

$$V_{cl} = f_{mes}(1 - \theta_{air})V_{leaf} \quad (10)$$

combining equations (9) and (10) gives:

$$S_{m,LA} = S_{m,Vcl}(1 - \theta_{air})f_{mes}L_{leaf} \quad (11)$$

Leaf mass per area (LMA) can be decomposed into the contribution of individual traits in a similar fashion as to the mesophyll surface area exposed to the airspace per leaf area, $S_{m,LA}$ (Eq. (1)), such that:

$$LMA = S_{m,LA}\frac{M}{V_{lf}}\frac{V_{lf}}{V_{cl}}\frac{1}{S_{m,Vcl}} \quad (12)$$

where $S_{m,LA}$ is the mesophyll surface area exposed to the intercellular airspace per leaf area, M is the leaf dry mass, V_{lf} is the total leaf cellular volume, computed from the total leaf volume minus the airspace volume, and V_{cl} is the mesophyll cell volume. Hence, M/V_{lf} is dry mass per total leaf cellular volume, i.e. the leaf cellular density, V_{lf}/V_{cl} is the ratio between the volumes of cells in the leaf and in the mesophyll, and $1/S_{m,Vcl}$ is the

volume of mesophyll cells per mesophyll cell surface exposed to the intercellular airspace. To compute the relative contribution to the change from shade to sun leaves, we computed LMA from the mean trait values, and mean trait values ± one standard deviation as in the methods presented in the main text. We then used the same methods presented for Eq. (4) and Eq. (5), replacing the traits in those equations with LMA (for Eq. (4)) and the traits from Eq. (12) (for Eq. (5)).

Results and discussion

Figure S6 presents the contribution of individual traits to the variation in LMA. $S_{m,LA}$ and M/V_{cl} were the most important contributors to LMA; both contributing up to ~50% of the total difference in LMA between shade and sun leaves. The other two traits had limited influence on LMA. The ratio of total leaf cellular volume per mesophyll cell volume (V_{lf}/V_{cl}) contributed to slightly lower LMA in sun leaves. Finally, the volume of mesophyll cells per leaf area ($S_{m,Vcl}$), which was substantially larger in sun leaves, does not contribute much to the differences in LMA (which was not the case for its contribution to $S_{m,LA}$).

Given that $S_{m,LA}$ is one of the two most important contributors to the differences in LMA, traits from Eq. (1) also contribute strongly to LMA, with leaf thickness being the highest contributor, as well as mesophyll porosity. Not surprisingly, the leaf cellular density (M/V_{lf}) is another important contributor and points to variations in cell wall density or cell wall volume (e.g. Harwood *et al.* 2021). Hence, within our model, LMA variation between sun and shade leaves is mainly driven by leaf thickness and density, the latter being driven by the cell wall volume per mesophyll volume, which is affected by many factors such as cellular density, mesophyll porosity, and/or cell size (e.g. Théroux-Rancourt *et al.* 2021).

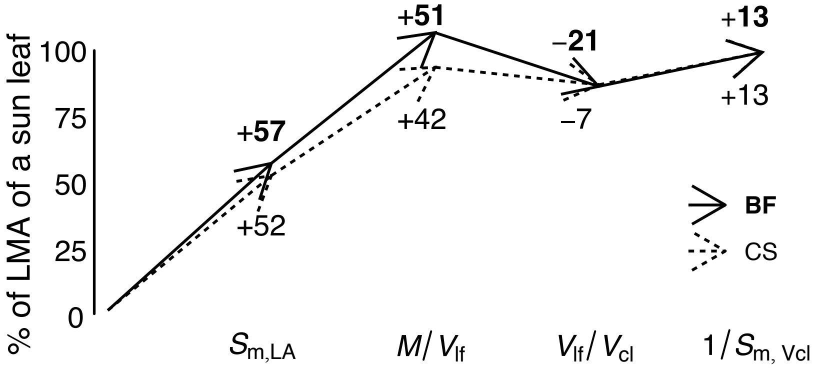

Figure S6: Contribution of individual traits to the difference in LMA between shade and sun leaves in Blaufränkisch (BF) and Cabernet Sauvignon (CS). Variable names have been shortened for figure clarity: $S_{m,LA}$: exposed mesophyll cell surface area per leaf area; M/V_{cl}: leaf dry mass per leaf cell volume; V_{lf}/V_{cl}: volume of cells within the leaf (V_{leaf} - V_{ias}) per mesophyll cell volume; 1 / $S_{m,Vcl}$: mesophyll cell volume per exposed mesophyll surface area.

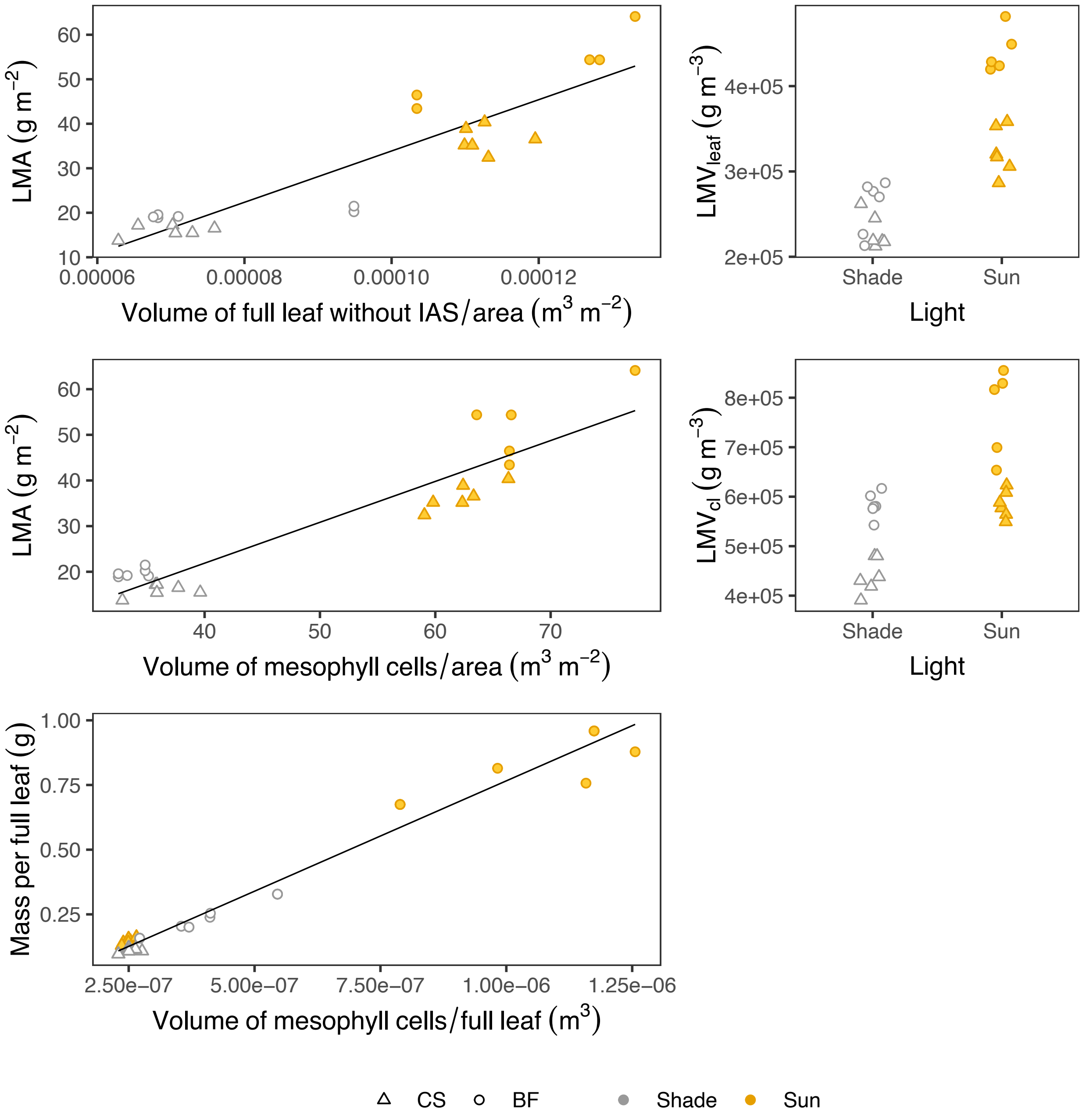

Figure S7: Relationship between leaf mass per area (LMA) and the extracted leaf cellular volume (total leaf volume minus airspace volume) or the mesophyll cell volume (left column). The difference in leaf mass per total leaf cellular volume (LMV_{leaf}) and per mesophyll cell volume (LMV_{cl}) between shade and sun leaves shows similar trends as for LMA (Figure S1). Six leaves per cultivar and light treatment were measured for both anatomy and LMA, except for BF shade plants for which only five plants were measured.

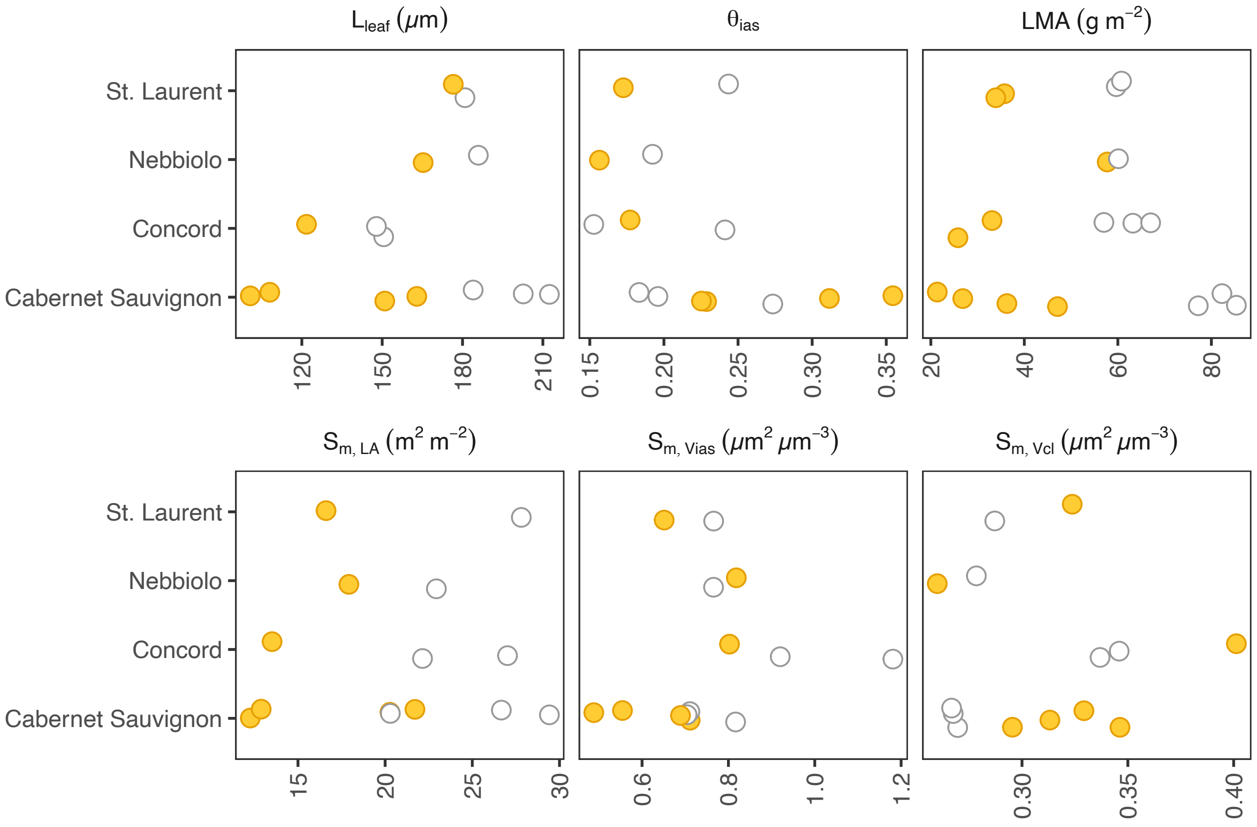

Figure S8: Anatomical values for vineyard-grown grapevine cultivars (5 years old plants) for shade (60% light transmission reduction; gray open) and sun grown leaves (orange filled). We see a substantial diversity in the response to shading in leaf thickness (L_{leaf}), $S_{m,LA}$, $S_{m,V}$ ratios, or porosity (θ_{ias}). However, leaf mass per area (LMA) is a trait in which shade leaves exhibit lower values, except for the measured leaves of the Nebiollo cultivar. Each point represents one leaf, with no replication per leaf.
